# Integrative single-cell and bulk transcriptomic analysis identify R-loop-associated gene signatures driving immune-inflammatory networks in acute pancreatitis

**DOI:** 10.3389/fimmu.2026.1946699

**Published:** 2026-09-29

**Authors:** Lujuan Pan, Weibang Wu, Tianying Zhang, Shujie He, Zujian Zhang, Cailing Zhang, Chaojun Lan, Yueqiu Qin

**Affiliations:** 1 Department of Gastroenterology, Affiliated Hospital of Youjiang Medical University for Nationalities, Baise, Guangxi, China; 2 Graduate School, Youjiang Medical University for Nationalities, Baise, Guangxi, China; 3 Guangxi Clinical Medical Research Center for Hepatobiliary Diseases, Affiliated Hospital of Youjiang Medical University for Nationalities, Baise, Guangxi, China; 4 Guangxi Zhuang Autonomous Region Engineering Research Center of Intelligent Biomedicine for Gastrointestinal Microecology, Affiliated Hospital of Youjiang Medical University for Nationalities, Baise, Guangxi, China

**Keywords:** acute pancreatitis, fibroblasts, inflammation, machine learning, MYL12A, R-loop

## Abstract

**Background:**

R-loop dysregulation has been implicated in chronic inflammation via DNA damage and cytoplasmic ssDNA release, yet its role in acute pancreatitis (AP) remains to be systematically investigated. This study aimed to identify R-loop-related biomarkers in AP and elucidate their potential roles in immune-inflammatory networks through integrative bioinformatics analysis and *in vitro* experimental validation.

**Methods:**

Single-cell and bulk transcriptomic datasets from the GEO database were integrated to calculate R-loop-related gene (RLRG) scores across cell types using multiple scoring algorithms. High-dimensional weighted gene co-expression network analysis (hdWGCNA), differential expression analysis, and machine learning algorithms (LASSO, SVM-RFE, and Boruta) were applied to screen candidate gene signatures. Immune infiltration and functional enrichment analyses were performed to investigate the regulatory mechanisms of identified gene signatures. A caerulein-induced AR42J cell model was established for experimental validation, and siRNA-mediated knockdown was conducted to assess functional roles.

**Results:**

Fibroblasts emerged as the primary cell type with elevated RLRG scores and significant differences between AP and control groups. Pseudotime analysis revealed that AP promotes fibroblast progression toward terminal differentiation, with high-RLRG-score cells exhibiting greater stemness and intercellular communication activity. Three candidate gene signatures—Anp32b, Myl12a, and Nras—were identified and showed markedly increased expression in the AP cohort. These genes exhibited significant correlations with immune cell populations and were enriched in immune-inflammatory, apoptotic, cytoskeletal, and proteasome pathways. The upregulation of Anp32b and Myl12a was confirmed in the caerulein-induced AR42J cell model. siRNA-mediated Myl12a knockdown attenuated trypsin activation and reduced inflammatory cytokine release, while immunofluorescence staining with the S9.6 antibody demonstrated that Myl12a knockdown significantly reduced nuclear R-loop accumulation.

**Conclusions:**

These findings highlight R-loop-associated gene signatures and functionally validated Myl12a as plausible candidates for dissecting AP pathogenesis. This work provides mechanistic clues for AP-related immune-inflammatory, which need further researches to verify.

## 1 Introduction

Acute pancreatitis (AP) is an inflammatory condition of the pancreas marked by intense abdominal discomfort, increased levels of amylase and lipase, and/or organ dysfunction (1). Global epidemiological statistics indicate an overall incidence of 33.74 cases per 100,000 individuals, exhibiting considerable regional variation (2). Gallstones, alcohol abuse, and hypertriglyceridemia represent the primary etiologies of AP, among which gallstone-induced AP is the most prevalent worldwide (2–4). As an urgent gastrointestinal disease requiring hospitalization, AP management must be tailored according to disease severity, complication care, and correction of underlying triggering factors (5). Therefore, comprehensive investigations into the molecular mechanisms of AP and delineation of the principal regulatory pathways governing inflammatory activation, tissue damage, and systemic complications hold considerable clinical significance and research relevance: clarifying disease progression, identifying targets for early diagnosis and intervention, optimizing personalized treatment, and improving long-term patient outcomes.

R-loops are dynamic three-stranded nucleic acid structures prevalent in the mammalian genome, participating in diverse biological processes such as DNA repair, cell proliferation and differentiation, RNA transcription and processing, gene expression regulation, and DNA methylation (6–9). Aberrant accumulation of R-loops, triggered by DNA damage, results in the release of cytoplasmic single-stranded DNA (ssDNA) and the activation of the cGAS-STING pathway, thereby promoting chronic inflammation and tissue degradation (10). R-loop dysregulation has been implicated in multiple sterile inflammatory conditions, including neuroinflammation (11) and mitochondria-dependent epithelial necrosis that drives spontaneous intestinal inflammation (12). R-loop-drived genomic instability also shapes inflammatory signaling within the tumor microenvironment (13), and emerging evidence further connects perturbed R-loop homeostasis to pancreatic tissue injury in chronic pancreatitis (14). Of note, acute pancreatitis is hallmarked by acinar cell DNA damage, massive cytosolic nucleic acid release, and robust cGAS-STING-mediated sterile inflammation-pathological events that closely overlap with downstream consequences of pathological R-loop accumulation (15).

Despite growing appreciation for the correlation between R-loop and pancreatitis, the R-loop-associated gene signatures and key mediators governing AP pathogenesis remain poorly defined. No systematic studies have yet characterized R-loop-related biomarkers and their functional roles in AP. The present study aims to fill this knowledge gap. By combining multiple bioinformatics analysis and *in-vitro* experimental validation, we interrogated R-loop-relevant gene signatures and cellular heterogeneity in AP, and further explored the biological functions of candidate genes in pancreatic acinar cell injury. Our work seeks to yield novel insights into AP molecular pathogenesis and uncover potential biomarkers and therapeutic targets for this disease.

## 2 Materials and methods

### 2.1 Data source

The AP-related datasets were extracted from the Gene Expression Omnibus (GEO) database (https://www.ncbi.nlm.nih.gov/geo/). The details of these datasets, including platform information, sample sizes, and sources, are summarized in **Supplementary Table 1**. Among these datasets, GSE109227 and GSE65146 were merged as the training cohort. GSE121038 and GSE3644were combined as the validation cohort. GSE181276 and GSE198183 were combined as the single-cell cohort. Batch effects between datasets within each cohort were removed using the ComBat function of sva R package (v3.52.0) (16). Additionally, a total of 1,185 R-loop-related genes (RLRGs) were obtained from the published literature (17) (**Supplementary Table 2**).

### 2.2 Single-cell transcriptomic and R-loop heterogeneity analysis

Single-cell data processing was performed using the Seurat R package (v5.1.0) (18). Quality control criteria were as follows: genes expressed in at least three cells were retained; cells with 200-8,000 genes, 200-100,000 counts, and a mitochondrial gene proportion of less than 25% were retained. After standardization, the top 2,000 highly variable genes were identified by the FindVariableFeatures function. Principal components with statistical significance were determined by the JackStrawPlot function, and the top 30 were selected for subsequent analysis. Cells were clustered using the FindNeighbors and FindClusters functions (resolution = 0.1) and annotated by comparing cluster-specific gene expression with known marker genes from published literature (19, 20). Doublets were identified and filtered out using the DoubletFinder R package (v2.0.4) (21), and the proportion of each cell type in each sample was quantified.

Based on the expression of RLRGs, the RLRG score of each cell type was calculated via the AUCell, UCell, singscore, ssGSEA, and AddModuleScore algorithms through irGSEA R package (v3.3.2) (22), and their mean value was obtained. Differences in each score and mean score between AP and control groups were analyzed. The cell type with the highest RLRG score and significant intergroup difference was defined as the key cell type.

The developmental trajectory of the key cell type was analyzed using the Monocle R package (v2.26.0) (23). According to the median RLRG score, the key cell type was divided into high- and low-score groups, and stemness scores were evaluated using the CytoTRACE R package (v1.1.0) (24). RNA velocity analysis was performed using Velocyto package to predict cellular differentiation trajectories based on the ratio of spliced to unspliced mRNA. Cell-cell communication between key cells in different score groups and other cell types was analyzed using CellChat package (v1.6.1) (25), and specific ligand-receptor interactions and signaling pathways were identified.

### 2.3 High-dimensional weighted gene co-expression network analysis

Based on the single-cell data, hdWGCNA was conducted with the RLRG scores as the trait through hdWGCNA package (v0.3.03) (26). The SetupForWGCNA and SetDatExpr functions were used to construct the expression matrix based on the Seurat object. The optimal soft threshold was selected using the TestSoftPowers function. The gene co-expression network was constructed using the ConstructNetwork function. Modules with a Pearson correlation coefficient > 0.5 to the trait in the key cell type were defined as key modules, and genes in these modules were considered key module-related genes.

### 2.4 Differentially expressed analysis

In the training cohort, differentially expressed genes (DEGs) between AP and control groups were identified using the limma R package (v3.54.2) (27), with |log_2_fold change (FC)| > 1 and adjusted *P*-value < 0.05 as thresholds. Results were visualized using volcano plots and heatmaps generated by the ggplot2 R package (v3.5.1) (https://ggplot2.tidyverse.org/).

### 2.5 Functional enrichment analysis

The intersection genes between DEGs and key module-related genes was obtained using the ggvenn R package (v0.1.10) (https://CRAN.R-project.org/package=ggvenn). Functional enrichment analysis of these intersection genes was performed using the clusterProfiler R package (v4.6.2), including Gene Ontology (GO) and Kyoto Encyclopedia of Genes and Genomes (KEGG) (28). GO terms or KEGG pathways with an adjusted *P*-value < 0.05 were considered statistically significant. Gene set enrichment analysis (GSEA) was conducted using the clusterProfiler R package with HALLMARK and KEGG gene sets from the MSigDB database downloaded via the Msigdbr R package. Pathways with |normalized enrichment score (NES)| > 1 and adjusted *P*-value < 0.05 were considered significant. Gene set variation analysis (GSVA) was performed using the GSVA R package (v1.46.0) (29) to calculate pathway scores for each sample in the training cohort, and differences between AP and control groups were compared using the limma R package. Pathways with |t| > 2 and *P*-value < 0.05 were considered significantly activated or inhibited.

### 2.6 Machine learning-based gene signature screening

The expression of intersection genes was analyzed in the training and validation cohorts, and differences between AP and control groups were compared. Genes with significant differences and consistent expression trends in both cohorts were selected as candidate biomarkers. Three machine learning algorithms, least absolute shrinkage and selection operator (LASSO), support vector machine-recursive feature elimination (SVM-RFE), and Boruta, were applied to identify feature genes. LASSO was performed using the glmnet R package (v4.1-8) (30), and the optimal lambda was determined by ten-fold cross-validation. SVM-RFE was performed using the e1071 R package (v1.7-14) (31) to evaluate and rank gene importance by iteratively calculating cross-validation error rates and accuracy for each feature combination, with the lowest-error combination selected as optimal. Boruta was used to identify the most relevant variables by comparing each feature with its randomly shuffled counterpart and eliminating those weakly correlated with the target variable through Boruta R package (v8.0.0) (32). Feature genes from the three algorithms were intersected, and the shared genes were defined as candidate gene signatures. The expression of these genes in each cell type was further investigated.

### 2.7 Immune infiltration analysis

The infiltration abundance of 33 immune cells in AP and control groups was evaluated using the ImmuCellAImouse R package (v0.1.0) (33), and differences between groups were analyzed by the Wilcoxon test. The associations between candidate gene signatures and immune cells were calculated using the Spearman algorithm.

### 2.8 Regulatory network construction

Transcription factors (TFs) interacting with candidate gene signatures were predicted using the ChEA3 database, and TFs supported by ChIP-seq data in the ENCODE database were retained (*P*-value < 0.05). miRNAs regulating candidate gene signatures were predicted using the miRDB database. lncRNAs interacting with predicted miRNAs were identified using the starBase database (clipExpNum > 5). TF-mRNA and lncRNA-miRNA-mRNA regulatory networks were constructed accordingly.

### 2.9 Drug prediction and molecular docking

Drugs interacting with candidate gene signatures were predicted using the DsigDB (https://coremine.com/medical/). The three-dimensional (3D) structures of candidate gene signatures were obtained from the UniProt database (http://www.uniprot.org), and drug structures were downloaded from the PubChem database (http://pubchem.ncbi.nlm.nih.gov/). Water molecules and small ligands were removed using AutoDock, and molecular docking was performed using AutoDock Vina. Results were visualized using PyMOL (34).

### 2.10 Cell culture and acute pancreatitis model induction

Pancreatic AR42J cells (cells of the rat exocrine pancreas, IM-R015) were purchased from the Immocell Biotechnology (Xiamen, China). Cells were cultured in F12K medium supplemented with 20% fetal bovine serum (FBS) and 1% penicillin-streptomycin (PS) at 37 °C in a humidified incubator with 5% CO_2_. The AP model was induced by stimulating cells with 100 nM caerulein for 24 hours (35).

### 2.11 Biochemical assays

The α-amylase level and trypsin activation were examined using α-amylase assay kit (C016-1-2-96t) and trypsin assay kit (A080-2-2), respectively, purchased from Nanjing Jiancheng Institute of Bioengineering (Nanjing, China). IL-6 and TNF-α levels were measured using ELISA kits (IL-6: ER1393; TNF-α: ER0042; FineTest, China). All procedures were performed according to the manufacturer’s instructions.

### 2.12 siRNA-mediated Myl12a knockdown in AR42J cells

Myl12a-silenced AR42J cells were established by transfecting cells with Myl12a-specific siRNA using RNATransMate reagent. Briefly, 140 pmol of siRNA and 10 μL of RNATransMate were each diluted in serum-free medium, mixed, and incubated at room temperature for 10 min to form siRNA/RNATransMate complexes. The mixture was added dropwise to cells seeded in 6-well plates at a density of 6 × 10^4^ cells per well in 2 mL complete medium. After 4–6 h, the transfection medium was replaced with fresh complete medium. Knockdown efficiency was validated by reverse transcription quantitative polymerase chain reaction (RT-qPCR).

### 2.13 RT-qPCR

Total RNA was extracted using the FastPure Complex Tissue/Cell Total RNA Isolation Kit (Vazyme, RC113-01) and reverse transcribed into cDNA using the ABScript III RT Master Mix for RT-qPCR with gDNA Remover (Abclonal, RK20429). Gene expression was quantified using the Genious 2X SYBR Green Fast RT-qPCR Mix (Abclonal, RK21205). mRNA levels were normalized to Gapdh as the internal reference. The relative expression was evaluated by the 2^-ΔΔCT^ method. Primers are listed in **Supplementary Table 3**.

### 2.14 Immunofluorescence staining

Cells grown on coverslips were gently washed with pre-chilled PBS, fixed with 4% paraformaldehyde for 15–20 min at room temperature, and permeabilized with 0.1% Triton X-100 for 10 min. Samples were then divided into two groups: the RNase H-treated group was incubated with 1–2 U/μL RNase H (D7089, Beyotime) at 37 °C for 30–45 min to degrade R-loops as a specificity control, while the control group was incubated with RNase H reaction buffer alone. After blocking with 5% BSA for 1 h, all groups were incubated overnight at 4 °C with S9.6 monoclonal antibody (1:200-1:500, YP562013, Abinscience), followed by secondary antibody incubation for 1 h in the dark. Negative controls were established by omitting the primary antibody or using an isotype IgG. After DAPI counterstaining, coverslips were mounted with anti-fade mounting medium, and images were captured using a fluorescence microscope.

### 2.15 Statistical analysis

Statistical analyses were performed using R software (v4.5.1) and GraphPad Prism. The Wilcoxon test was utilized to assess differences between two groups in the bioinformatics study. The Student’s t-test was applied to analyze the experimental data. P < 0.05 was considered statistically significant. For multiple-testing correction, the Benjamini-Hochberg false discovery rate (FDR) method was applied to adjust raw P-values. Features with FDR-adjusted P-value < 0.05 were considered statistically significant.

## 3 Results

### 3.1 Single-cell transcriptomic profiling identifies fibroblasts as key cells

After quality control, 18 clusters were obtained through the cluster analysis and were annotated to 11 cell types: endocrine cells, red blood cells, stellate cells, fibroblasts, endothelial cells, mesothelial cells, ductal cells, acinar cells, macrophages, B cells, and T cells (**Figures 1A, B**). After doublet removal, cell boundaries were clearer, indicating effective reduction of technical noise (**Figure 1C**). In the control group, acinar cells were the most abundant, whereas fibroblasts were the most abundant in the AP group (**Figure 1D**). Among these cell types, fibroblasts exhibited the highest RLRG score with significant differences between AP and control groups (**Figure 1E**). Therefore, fibroblasts were defined as key cells.

**Figure 1:**
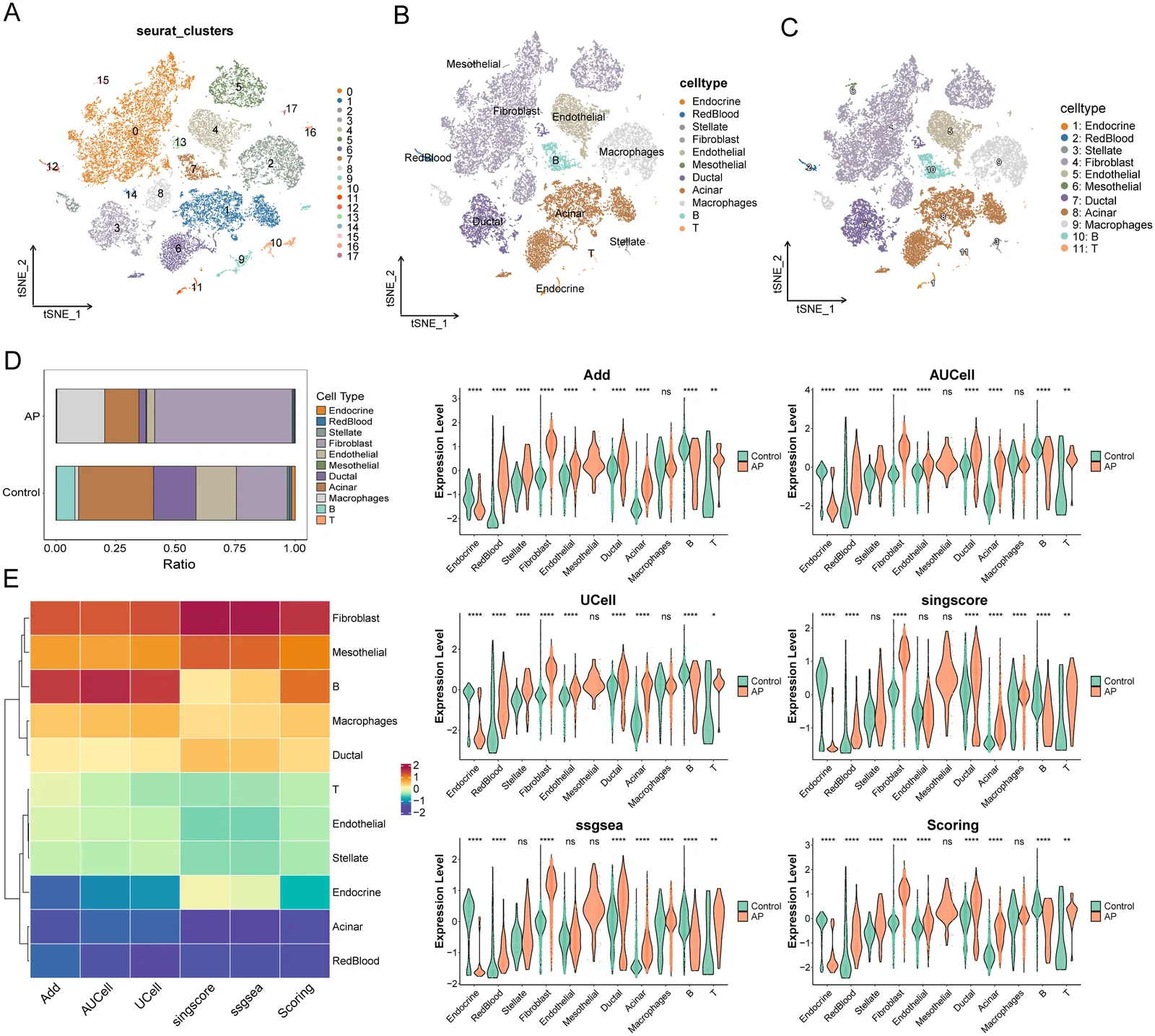
Single-cell transcriptomic landscape and multi-algorithm R-loop-related gene scoring in acute pancreatitis. **(A)** t-Distributed Stochastic Neighbor Embedding (tSNE) visualization of 18 transcriptional cell clusters. **(B)** tSNE shows the distribution of 11 cell types. **(C)** tSNE projection after doublet removal. **(D)** Proportion of each cell type in AP and control samples. **(E)** R-loop-related gene (RLRG) scores across cell types computed by five algorithms. Left: heatmap of RLRG scores per cell type; Right: differential RLRG scores between AP and control groups. ns: not significant, *P < 0.05, **P < 0.01, ***P < 0.001, ****P < 0.0001.

Pseudotime analysis revealed that fibroblasts undergo continuous state evolution along an unbranched trajectory during AP, with AP promoting progression toward terminal differentiation (**Figure 2A**). High RLRG score identified a less differentiated, stem-like subpopulation at early stages, while low RLRG score marked terminally differentiated cells, consistent with the higher stemness score in the high-score group (**Figure 2B**). In contrast, RNA velocity analysis showed that fibroblasts exhibited short, scattered velocity vectors with limited directional flow (**Figure 2C**), indicating that fibroblasts maintained a relatively stable transcriptional state without active lineage transition, while retaining potential for further state evolution.

**Figure 2:**
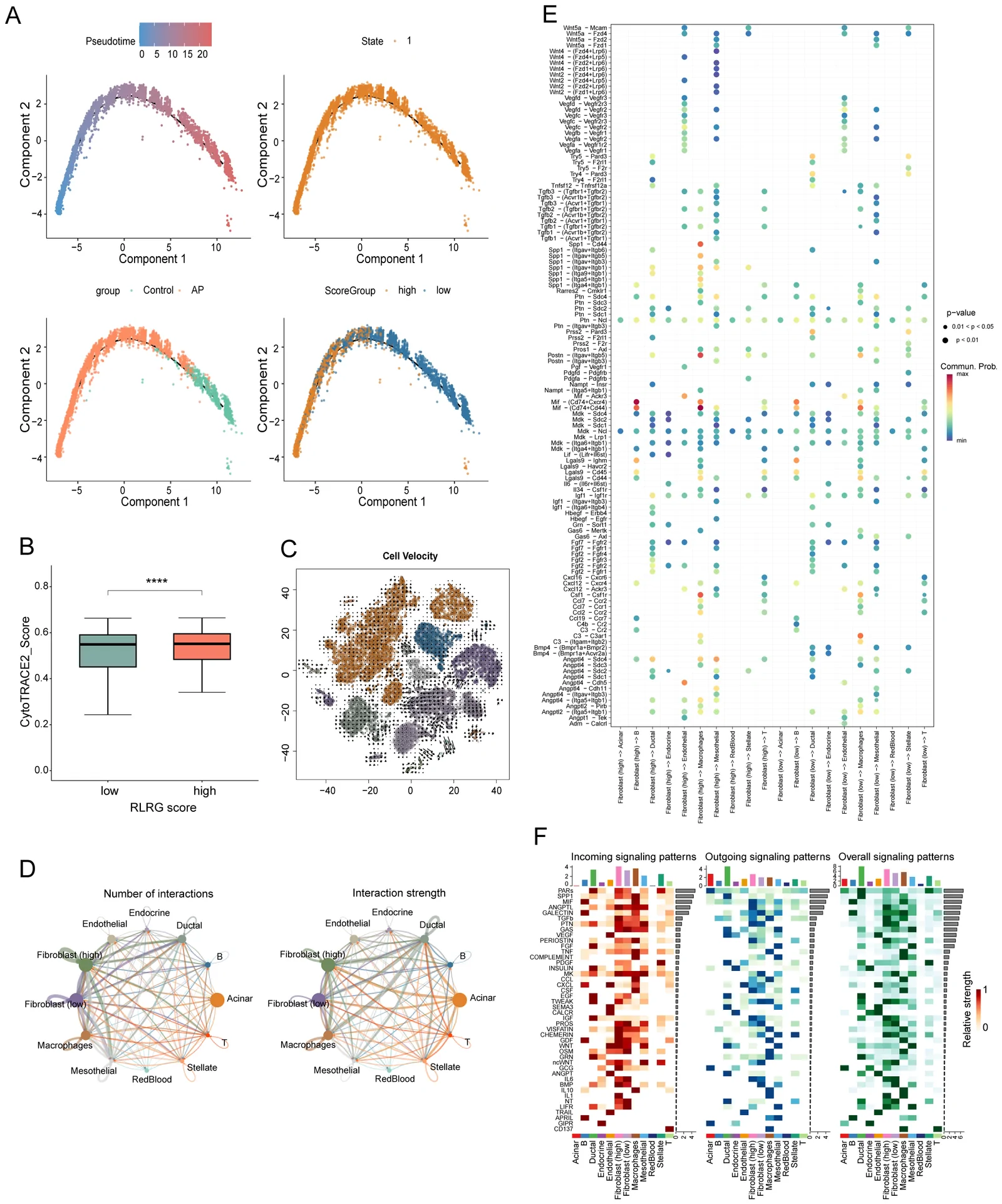
Pseudotemporal trajectory and intercellular communication dynamics of high-RLRG-score fibroblasts. **(A)** Pseudotime analysis of fibroblasts. From left to right: cells colored by pseudotime value; by differentiation stage; by different samples; and by RLRG score. **(B)** CytoTRACE analysis of stemness scores between high- and low-RLRG-score groups. **(C)** RNA velocity analysis. **(D)** Cellular communication network showing interaction number and strength across cell types. **(E)** Ligand-receptor interaction networks in high- and low-score groups. **(F)** Signaling pathway activity across cell types. ****P < 0.0001.

Cell-cell communication analysis demonstrated that high-score fibroblasts exhibited more interactions and greater interaction strength with other cell types compared to the low-score group (**Figure 2D**). In high-score fibroblasts, key interaction pairs included Mif-(Cd74+Cxcr4) (fibroblasts → B cells), Mif-(Cd74+Cd44) (fibroblasts → macrophages), and Csf1-Csf1r (fibroblasts → macrophages) (**Figure 2E**). Signaling pathway analysis revealed stronger outgoing and incoming signaling activities in high-score fibroblasts, particularly in MIF, SPP1, PTN, TGFb, and MK pathways (**Figure 2F**), suggesting their potential contribution to intercellular communication.

### 3.2 Machine learning-based identification of candidate gene signatures

hdWGCNA identified four gene modules correlated with the RLRG score of fibroblasts (correlation coefficient > 0.5): green (Cor = 0.863), red (Cor = 0.831), grey60 (Cor = 0.709), and magenta (Cor = 0.612) (**Figure 3A**). Therefore, these four modules were key modules, and 1,010 key module-related genes were obtained. Differential expression analysis identified 1,006 DEGs between AP and control groups, including 903 upregulated and 103 downregulated genes (**Figure 3B**). The intersection of these two gene sets yielded 79 genes, which were significantly enriched in 506 GO terms and 26 KEGG pathways (**Supplementary Table 4**), such as actin filament organization, adherens junction, actin binding, and regulation of actin cytoskeleton (**Figures 3C, D**). Among these 79 genes, 38 showed significant differential expression and consistent trends in both training and validation cohorts (**Supplementary Table 5**). Thereafter, LASSO, SVM-RFE, and Boruta identified 4, 15, and 31 feature genes, respectively (**Figures 4A–C**). Three shared genes were identified: Anp32b, Myl12a, and Nras, all significantly upregulated in the AP group (**Figures 4D, E**). These were defined as candidate gene signatures. These three genes showed markedly different expression between AP and control groups in fibroblasts, endocrine cells, and acinar cells (**Figure 4F**), with higher expression at the trajectory origin of fibroblast differentiation (**Figure 4G**). These findings further confirm that Anp32b, Myl12a, and Nras are specifically enriched in fibroblasts. Along the fibroblast pseudotime trajectory, these genes exhibit higher expression at the trajectory origin, with expression gradually declining at differentiation progresses. These observations further highlight the potential involvement of fibroblasts and these candidate gene signatures in AP pathogenesis.

**Figure 3:**
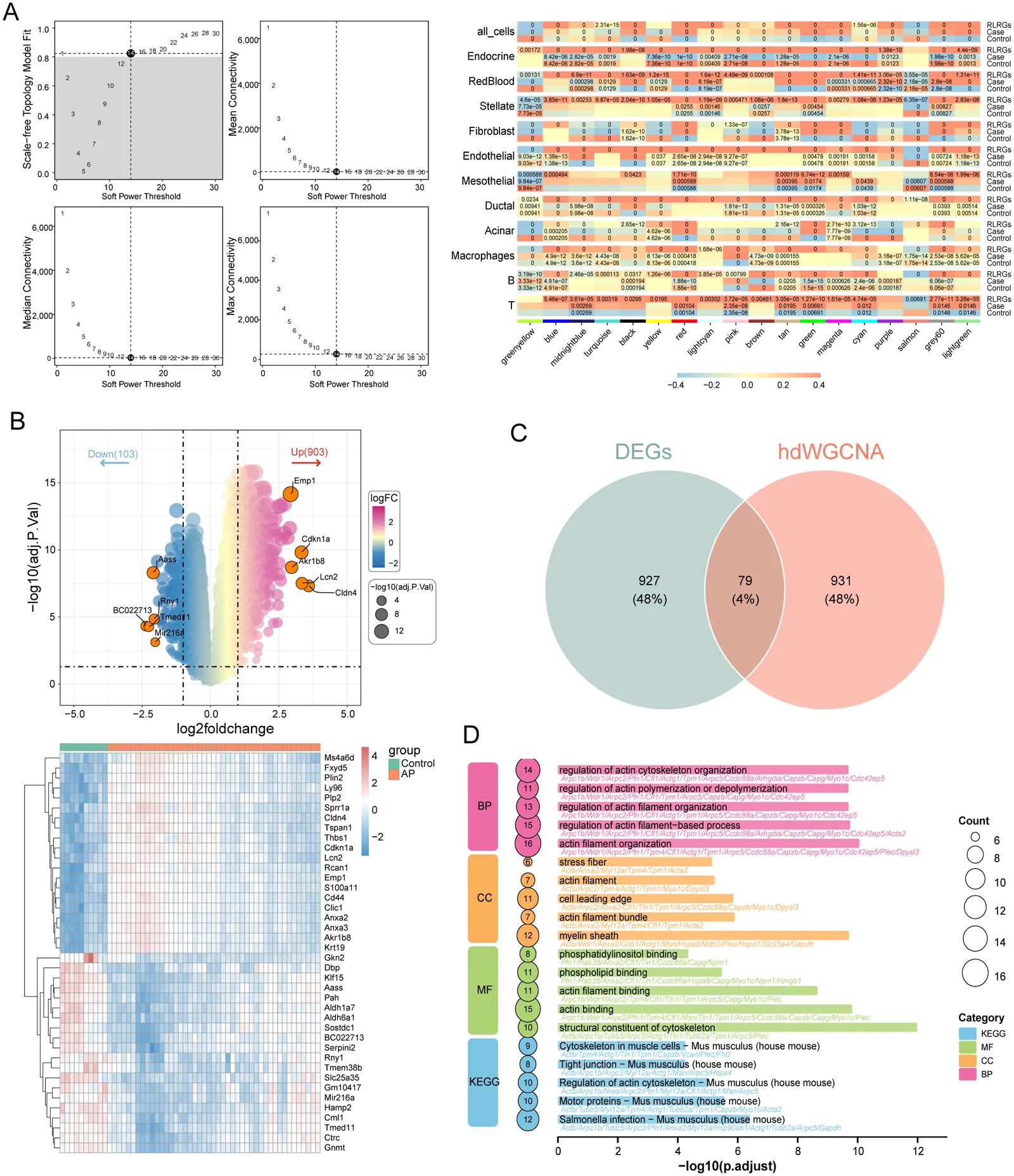
Integrative hdWGCNA and differential expression analysis identifies core genes and functional enrichment analysis. **(A)** High-dimensional weighted gene co-expression network analysis (hdWGCNA). Left: determination of optimal soft threshold; Right: module-trait correlations across cell types. **(B)** DEGs between AP and control groups. Top: volcano plot; Bottom: heatmap. **(C)** Intersection of key module-related genes and DEGs. **(D)** Functional enrichment analysis of intersection genes.

**Figure 4:**
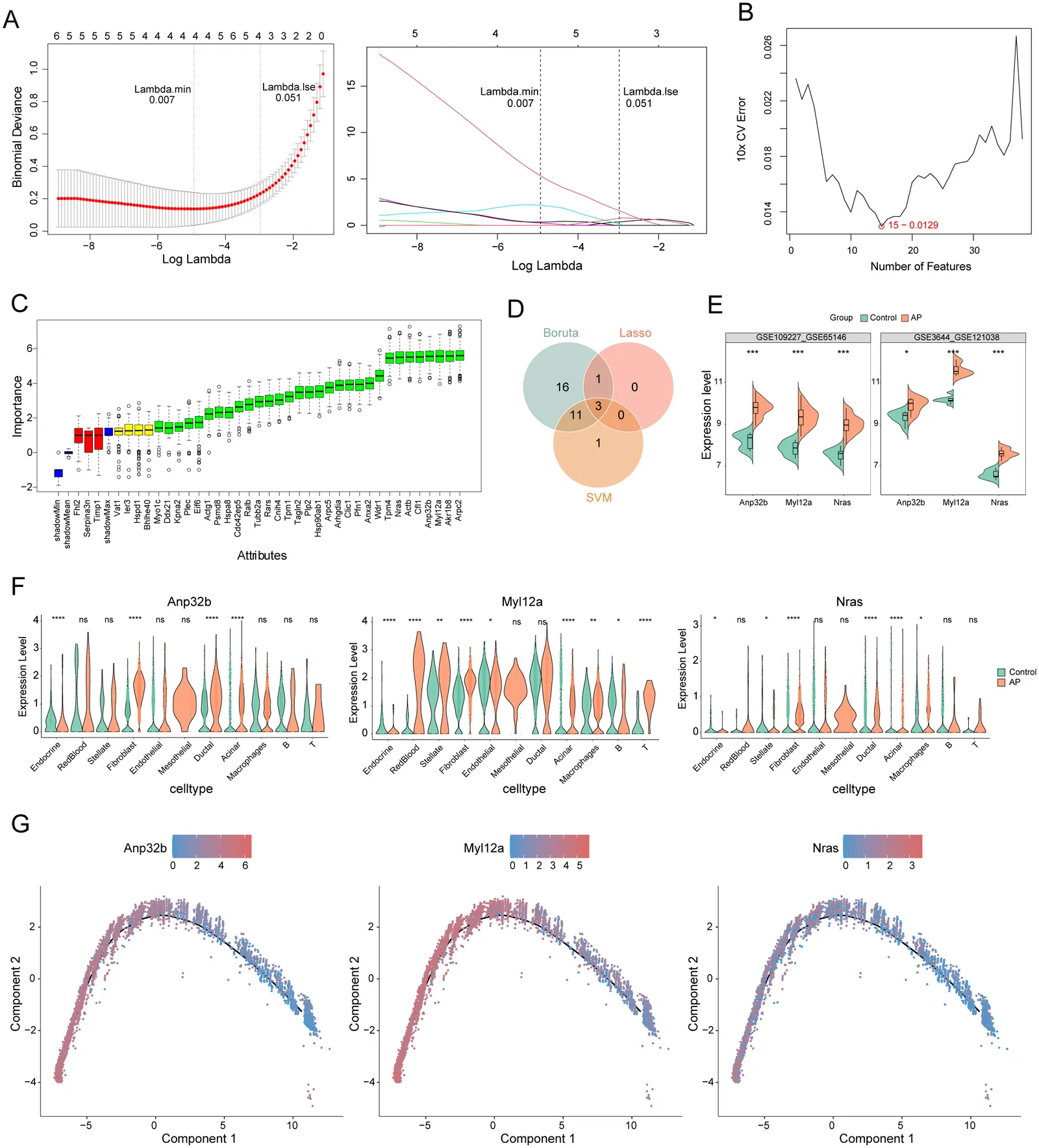
Machine learning-driven identification of R-loop-related candidate gene signatures. **(A)** Least absolute shrinkage and selection operator (LASSO). **(B)** Support vector machine-recursive feature elimination (SVM-RFE). **(C)** Boruta algorithm. **(D)** Venn diagram of characteristic genes from three algorithms. **(E)** The expression of candidate gene signatures in training and validation cohorts. **(F)** The expression of candidate gene signatures in different cell types. **(G)** Candidate gene signature expression dynamics during fibroblast differentiation. ns, not significant; *P < 0.05, **P < 0.01, ***P < 0.001, ****P < 0.0001.

### 3.3 Immune microenvironment and candidate gene signatures associations

To further investigate the relationship between the identified candidate gene signatures and immune status, immune infiltration analysis revealed the abundance of 33 immune cells across samples (**Figure 5A**). Among these, 28 cell types showed significantly altered infiltration between AP and control groups, including B cells, dendritic cells, and macrophages (**Figure 5B**). Correlation analysis demonstrated marked correlations between three candidate gene signatures and these immune cell populations (**Figure 5C**). Myl12a showed the strongest positive correlation with macrophages (Cor = 0.912, *P* < 0.001) and the strongest negative correlation with natural killer cells (Cor = -0.819, *P* < 0.001). These results indicate that the identified candidate gene signatures are closely associated with immune cell infiltration, suggesting their potential involvement in the immunopathogenesis of AP.

**Figure 5:**
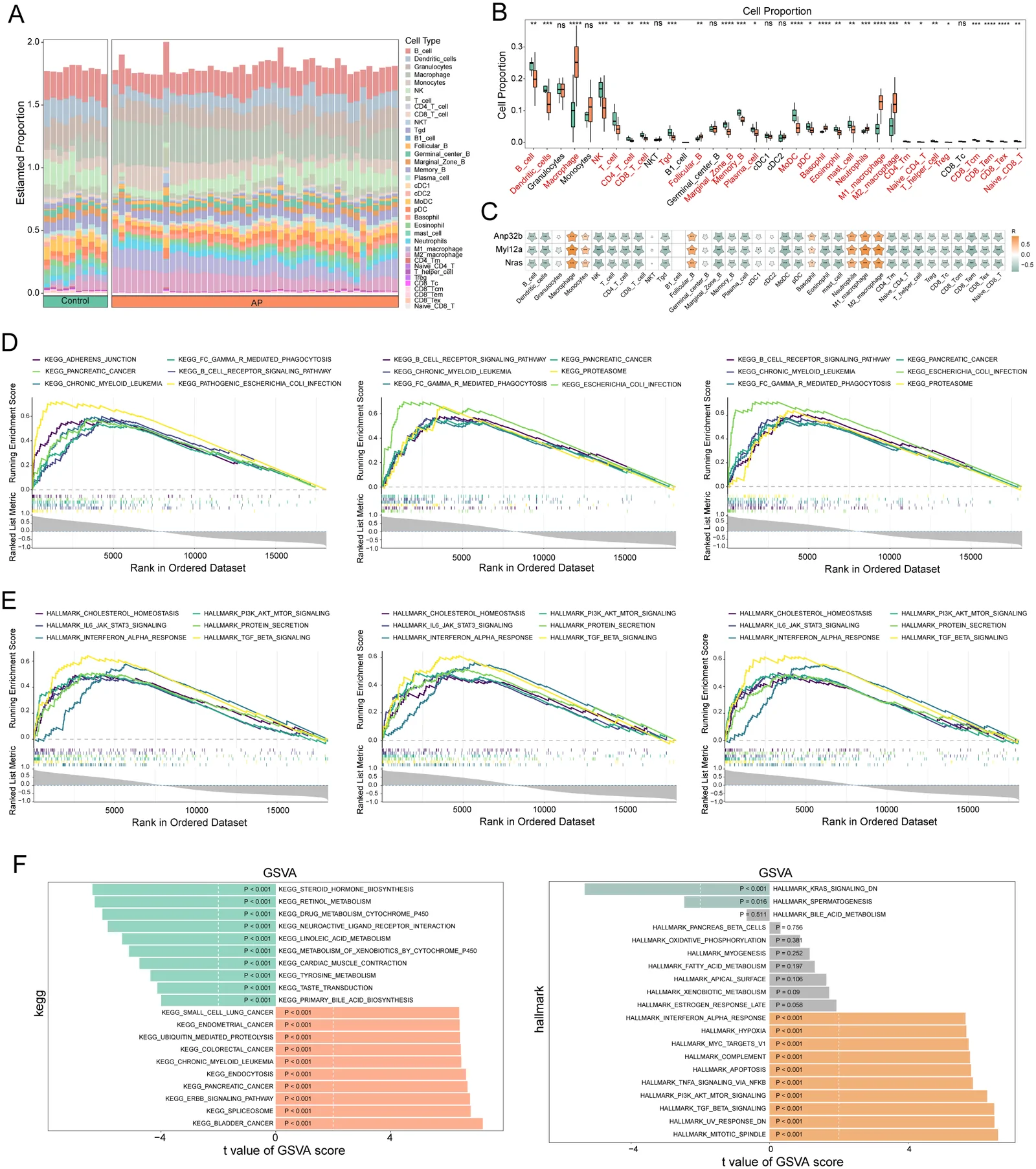
Immune infiltration profiling and functional enrichment analyses. **(A-C)** Immune infiltration analysis. **(A)** Infiltration abundance of 33 immune cells across samples. **(B)** Different infiltration abundance of immune cells between AP and control groups. **(C)** Correlation heatmap of candidate gene signatures with immune cells. **(D, E)** Gene set enrichment analysis (GSEA) analysis. **(D)** GSEA with KEGG gene sets as reference sets. **(E)** GSEA with HALLMARK gene sets as reference sets. From left to right: Anp32b, Myl12a, and Nras. **(F)** Gene set variation analysis (GSVA). From left to right: GSVA with KEGG gene sets as reference sets; GSVA with HALLMARK gene sets as reference sets. ns, not significant; *P < 0.05, **P < 0.01, ***P < 0.001, ****P < 0.0001.

### 3.4 Functional enrichment of candidate gene signatures

To further investigate the mechanisms by which candidate gene signatures regulate AP pathogenesis, GSEA was conducted. With the KEGG gene set as the reference, Anp32b, Myl12a, and Nras were significantly associated with 71, 70, and 70 pathways, respectively (**Supplementary Table 6**). With the HALLMARK gene set as the reference, they were significantly associated with 11, 10, and 10 pathways, respectively (**Supplementary Table 6**). According to significance ranking, the top six pathways are shown (**Figures 5D, E**). These three candidate gene signatures shared functional similarities, being significantly associated with immune and inflammatory-related pathways (such as B cell receptor signaling pathway, NOD-like receptor signaling pathway, IL-6-JAK-STAT3 signaling), apoptosis and cell survival-related pathways (such as apoptosis, p53 signaling pathway, PI3K-AKT-mTOR signaling), cytoskeleton and cell adhesion-related pathways (such as adherens junction, ECM-receptor interaction), as well as proteasome and protein secretion-related pathways and metabolism-related pathways. These findings indicate that the candidate gene signatures may contribute to AP pathogenesis through multiple mechanisms, including immune dysregulation, impaired protein homeostasis, cytoskeletal disruption, and metabolic alterations.

### 3.5 GSVA reveals widespread dysregulation of pathways

GSVA analysis revealed that immune- and inflammation-related pathways, including B cell receptor signaling pathway, Toll-like receptor signaling, FcγR-mediated phagocytosis, NOD-like receptor signaling, and IL-6-JAK-STAT3 signaling, were significantly activated in AP (**Supplementary Table 7**, **Figure 5F**). Cytoskeleton and cell adhesion-related pathways (such as regulation of actin cytoskeleton, adherens junction, tight junction) and the proteasome pathway were also activated, while metabolic pathways such as valine, leucine and isoleucine degradation were suppressed.

### 3.6 Creation of regulatory networks, drug prediction, and molecular docking

After screening, 9 TFs were predicted (**Figure 6A**). Among these TFs, MAX, USF1, SRF and REST both had interactions with all candidate gene signatures. The lncRNA-miRNA-mRNA regulatory network contained 3 mRNAs, 36 miRNAs, and 8 lncRNAs, and the lncRNA-miRNA-mRNA regulatory pairs included Oip5os1-mmu-let-7c-5p-Nras, Meg3-mmu-miR-361-5p-Myl12a, and Mir17hg-mmu-miR-673-5p-Anp32b (**Figure 6B**). Subsequently, with candidate gene signatures as targets, a total of 29 drugs had interactions with them (**Figure 6C**). Among these drugs, vitamin E had interactions with Anp32b and Nras. Based on the significance, the interactions of dacarbazine-Nras, Clofarabine-Anp32b, and temozolomide-Myl12a were evaluated by molecular docking. Results showed that the binding of Clofarabine-Anp32b and temozolomide-Myl12a was relatively stable (binding energy: Clofarabine-Anp32b: -5.493 kcal/mol; temozolomide-Myl12a: -5.498 kcal/mol) (**Table 1**, **Figure 6D**).

**Figure 6:**
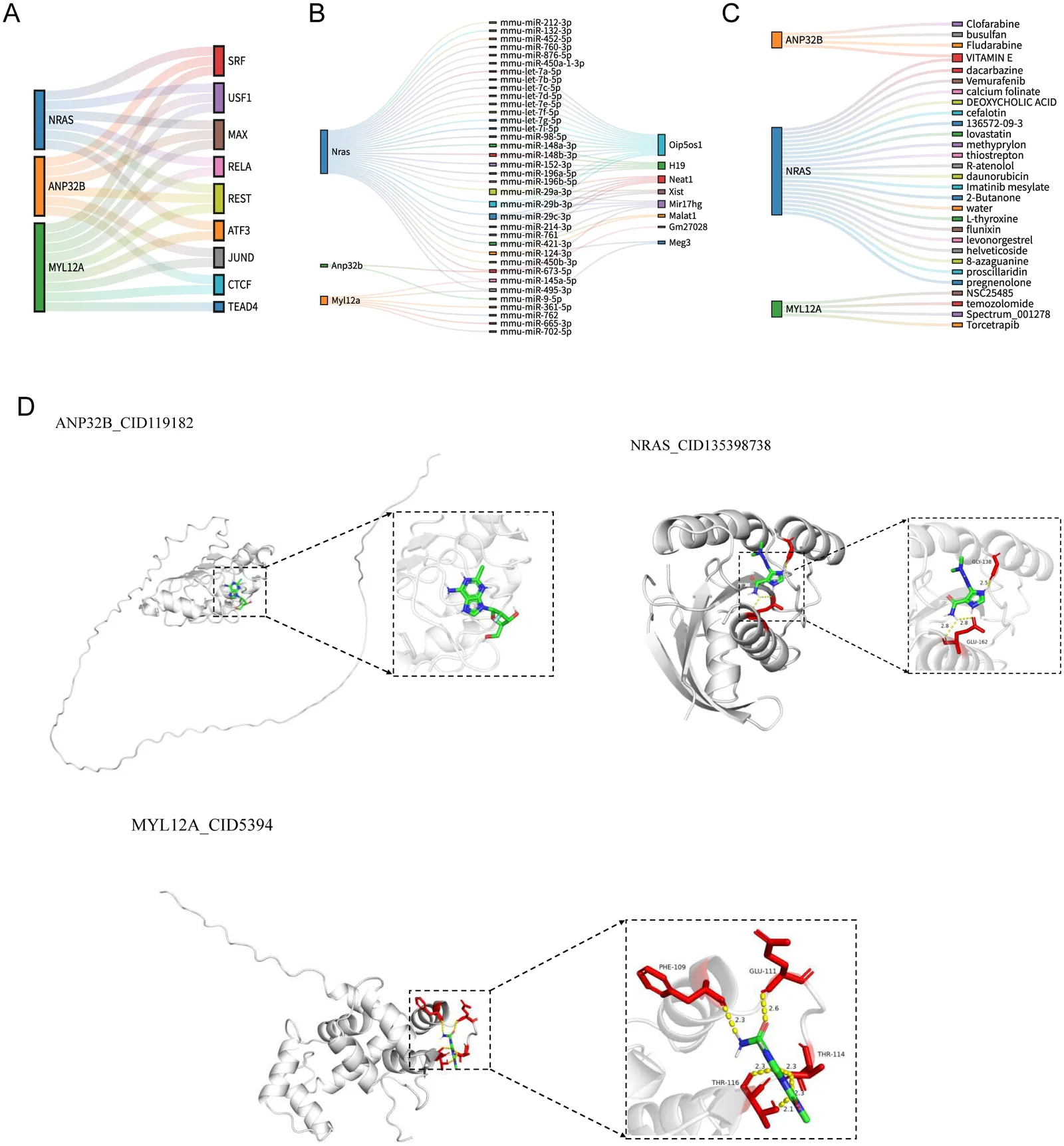
Integrative analysis of regulatory networks, drug prediction, and molecular docking. **(A)** TF-mRNA regulatory network. **(B)** lncRNA-miRNA-mRNA regulatory network. **(C)** Drug-mRNA network. **(D)** Molecular docking.

**Table 1:** Molecular docking.

Gene	UniProt	PDB	Drug	CID of drug	Binding energy	Number of hydrogen bonds
NRAS	P01111	3CON	dacarbazine	CID135398738	-4.18	3
ANP32B	Q92688	AF-Q92688-F1	Clofarabine	CID119182	-5.493	0
MYL12B	O14950	AF-O14950-F1	Spectrum_001278	CID5702210	-6.218	3

### 3.7 Expression and functional validation of Myl12a

To validate the role of candidate gene signatures in AP, an AP model was established in AR42J cells using caerulein stimulation. Elevated levels of α-amylase, IL-6, and TNF-α confirmed successful model induction (**Figures 7A–C**). Anp32b and Myl12a were significantly upregulated in the AP group compared to controls, consistent with bioinformatics findings, whereas Nras showed no significant difference (**Figure 7D**). Considering the prominent expression dynamics across fibroblast pseudotime trajectory and high confidence in our results, Myl12a was selected for subsequent functional analysis. Among three siRNAs, si1 and si3 significantly reduced Myl12a mRNA expression compared to siRNA negative control (si-nc), with si3 showing superior knockdown efficiency and selected for further experiments (**Figure 7E**). Trypsin activity was significantly elevated in the AP model group compared to controls, and Myl12a knockdown markedly reduced trypsin activity compared to the AP model group, suggesting attenuated pancreatic enzyme activation (**Figure 7F**). Furthermore, R-loop levels were significantly reduced following Myl12a knockdown compared to the AP model group (**Figure 7G**). Collectively, these findings demonstrate that Myl12a is upregulated in AP and that its knockdown alleviates pancreatic enzyme activation and reduces R-loop accumulation, suggesting a potential role in AP pathogenesis through R-loop regulation.

**Figure 7:**
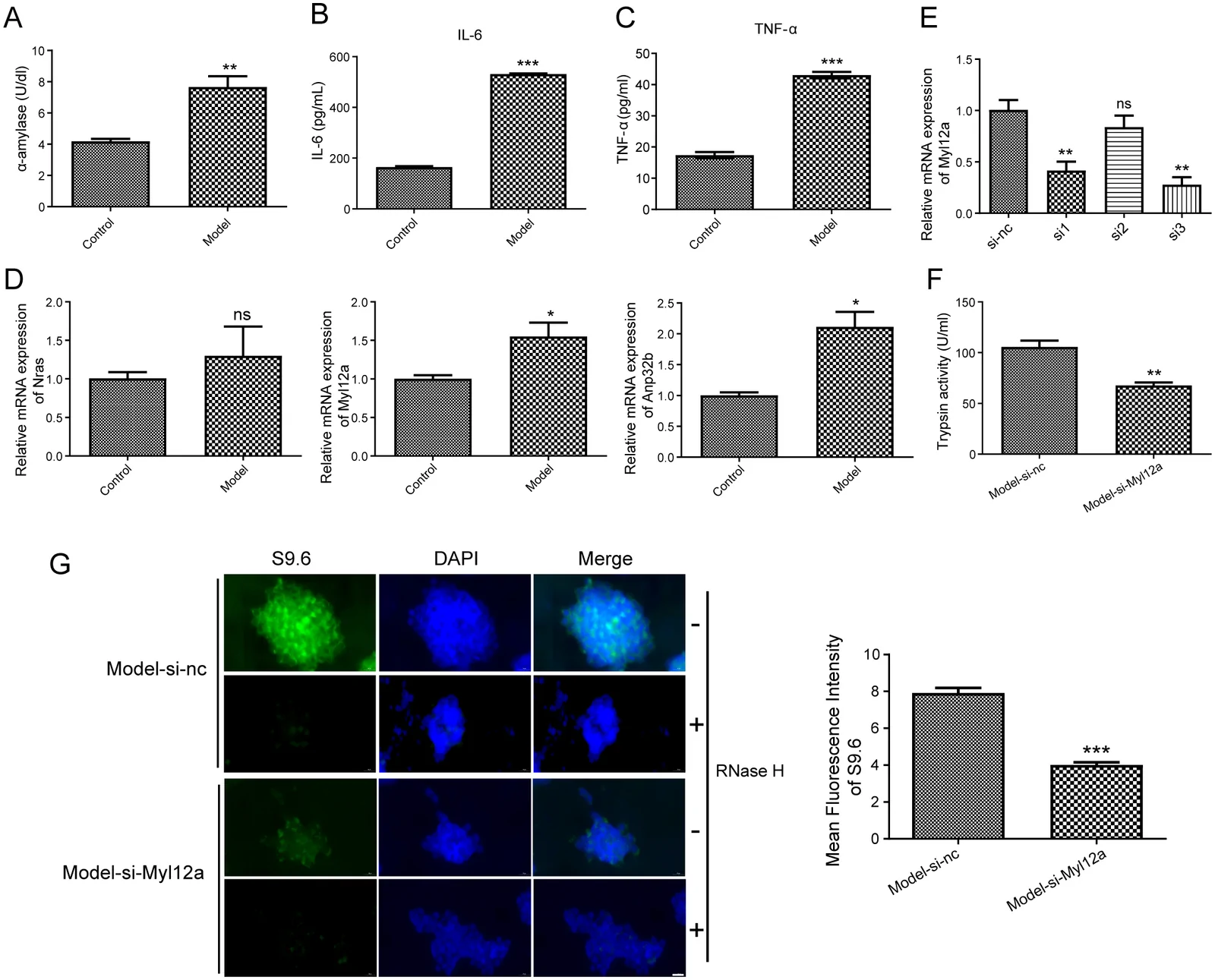
Functional validation of Myl12a *in vitro* acute pancreatitis model. **(A)** The α-amylase level between model and control groups. **(B)** The IL-6 level between model and control groups. **(C)** The TNF-α level between model and control groups. **(D)** The expression of candidate gene signatures between model and control groups. **(E)** Validation of Myl12a gene knockdown efficiency in different siRNA transfection groups. **(F)** The trypsin activity between model-si-Myl12a and model-si-nc groups. **(G)** Immunofluorescence staining of R-loops using S9.6 antibody (green) in acute pancreatitis model cells. Nucleus was stained with DAPI. Scale bar = 20 μm. ns: not significant, *P < 0.05, **P < 0.01, ***P < 0.001.

## 4 Discussion

Among patients with AP, roughly 20%–30% progress to moderate-to-severe pancreatitis, frequently associated with complications; in cases with infectious pancreatic necrosis, the death rate may ascend to 30% (36). Therefore, investigating the pathophysiology of AP will certainly enhance clinical comprehension for its diagnosis and management. R-loop dysregulation has been identified as correlated with replication stress and heightened DNA damage, physiological states associated with several diseases (37). Nevertheless, studies concerning the possible functions of R-loop-associated targets in AP are scarce, leading us to initiate a preliminary exploration of R-loops in AP by a integration of bioinformatics and experimental techniques. In our study, fibroblasts were identified as key cells linked to R-loops in the progression of AP.

Notably, fibroblasts, particularly pancreatic stellate cells, assume a crucial role that resembles a “double-edged sword” in AP. On the one hand, hypoxia stimulates fibroblasts to enhance glycolysis-dependent production of CXCL1 through the NF-κB-HIF-1α signaling pathway, attracting CXCR2-positive neutrophils to infiltrate the tissue and intensifying pancreatic inflammation (38). On the other hand, fibroblasts serve a crucial protective function—traditional *in vitro* and *in vivo* investigations have shown that during the regeneration phase of the AP, fibroblasts can markedly enhance acinar cell proliferation via paracrine secretion of insulin-like growth factor I (IGF-I), and fibroblast-conditioned medium can be partially inhibited by IGF-I antibodies, suggesting that IGF-I is a pivotal effector molecule facilitating tissue repair by fibroblasts (39). Meanwhile, animal studies indicate that the lack of fibroblast growth factor 21 markedly intensifies pancreatic tissue damage and elevates mortality rates in mice with acute pancreatitis; this factor may also ameliorate the pathological progression of acute pancreatitis by activating Sirt1-related signaling pathways, thereby clarifying the protective roles of fibroblast-related factors in acute pancreatitis (40–42). Importantly, Zhou et al. reported that lncRNA MEG3 was specifically down-regulated in cancer-associated fibroblasts (CAFs). The crosstalk between MEG3-deficient CAFs and tumor epithelial cells was strongly dependent on the TGF-β pathway, which consequently facilitated the epithelial-mesenchymal transition (EMT) program (43). Single-cell sequencing has uncovered the heterogeneity of fibroblasts in AP, revealing subpopulations, such as quiescent fibroblasts, myofibroblasts, and antigen-presenting fibroblasts, that undergo dynamic differentiation during disease progression, with some driving inflammation via chemokines like CXCL1 while others potentially participating in immune regulation and tissue repair (44). The aforementioned research has definitively shown that fibroblasts serve a dual regulatory function in the inflammatory damage and tissue repair linked to AP. In alignment with prior research, this study identified a notable increase in the proportion of fibroblasts within pancreatic tissue affected by AP and further confirmed that fibroblasts are a crucial cell population implicated in the initiation and advancement of AP, closely linked to the disruption of R-loop homeostasis.

Subsequently, three R-loop and fibroblast-associated candidate gene signatures—Anp32b, Myl12a, and Nras—were found, all of which were considerably elevated in AP. Accumulating experimental evidence supports the intrinsic biological links between our three candidate biomarkers and fibroblast function. Exosomes carrying ANP32B are functionally associated with type-I collagen biosynthesis in fibroblasts, as reported in a study focusing on dermal stem/progenitor-cell-derived exosomes (45). MYL12A is documented to mediate the contractile phenotype of fibroblasts (46). As for NRAS, prior work has demonstrated its regulatory roles in fibroblast activation, cell proliferation, and acquisition of profibrotic cellular features (47). The expression of Anp32b and Myl12a was confirmed in an *in vitro* AP cell model. Moreover, these candidate gene signatures exhibited a substantial correlation with immune cell infiltration and were prevalent in pathways including immunological inflammation, apoptosis, cytoskeletal remodeling, and proteasome activation, all of which jointly contribute to the pathogenesis of AP. ANP32B, or PAL31, is a constituent of the leucine-rich repeat (LRR) protein superfamily and is integral to cell proliferation, apoptosis, and cell cycle progression (48–50). Myl12a is a crucial regulatory member of the non-muscle myosin II complex, whose phosphorylation governs multiple cytoskeletal processes, including cell contraction, cytokinesis, cell migration, and stress fiber formation (51). A previous study has reported Myl12a as a fibroblast-associated prognostic gene in pancreatic cancer, implying its potential involvement in pancreatic stromal remodeling (52). In our *in vitro* AP cell model, siRNA-mediated Myl12a knockdown markedly reduced intracellular trypsin activity, suggesting that Myl12a may aggravate acinar cell injury and inflammatory responses, thus participating in pancreatic pathological alterations during AP progression. Clinically, intestinal barrier structural and functional defects are commonly observed in AP patients (53). Consistently, animal studies have further demonstrated that increased myosin light chain (MLC) phosphorylation is tightly linked to intestinal mucosal barrier disruption in severe AP (54). As a canonical MLC family protein and a downstream target of the RhoA/ROCK signaling axis (55), Myl12a may modulate non-muscle myosin II contractility. On this basis, it is plausible that Myl12a participates in acinar cell morphological remodeling, endothelial and intestinal epithelial junction destabilization, and leukocyte transendothelial migration, which may further contribute to AP-associated intestinal barrier dysfunction. NRAS, a member of the classic oncogenic RAS family, is significantly associated with the alteration of the inflammatory milieu (56, 57). Nevertheless, so far, no studies have thoroughly examined the expression alterations and biological roles of Anp32b, Myl12a, and Nras in AP, nor is there evidence demonstrating their regulatory relationship with the breakdown of R-loop homeostasis in AP. Accordingly, our study has first identified three candidate gene signatures associated with the fibroblast-derived RLRG score and R-loop in AP.

Immune infiltration analysis demonstrated robust correlations between all candidate gene signatures and immune cell populations. Functional enrichment analysis further uncovered prominent activation of immune-related pathways within the AP group. These candidate gene signatures were closely linked to multiple immune signaling cascades, including the B cell receptor signaling pathway, Toll-like receptor signaling, FcγR-mediated phagocytosis, NOD-like receptor signaling, and IL-6-JAK-STAT3 signaling. The pathways are pivotal in the inflammatory cascade of AP. Damage-associated molecular patterns (DAMPs) such as HMGB1, DNA, and ATP, released during acinar cell necrosis, are detected by Toll-like receptors (TLRs) and P2X7 receptors, which activate the NLRP3 inflammasome, facilitating the maturation and secretion of IL-1β and IL-18 (58, 59). IL-6 propagates local pancreatic damage and systemic inflammatory responses through the JAK/STAT3 signaling pathway (60). Recent findings indicate that tofacitinib enhances macrophage M1 polarization and mitigates the AP-induced inflammatory response by blocking the primary JAK/STAT signaling pathway and the developing NOD-like receptor signaling pathway (61). The stimulation of the B cell receptor signaling pathway indicates that B-cell-mediated adaptive immunity may contribute to AP, supplementing earlier views that predominantly emphasized innate immune cells (62). The substantial enhancement of FcγR-mediated phagocytosis pathways indicates that immune complex clearance and cellular effector activities may play a role in the immunological regulatory network of AP. Consequently, the candidate gene signatures discovered in this investigation may contribute to the initiation, amplification, and persistence of inflammation in acute pancreatitis through the synergistic modulation of these multifaceted immune pathways.

This work is the first to establish a functional linkage between R-loop and fibroblasts inAP. Through multidimensional bioinformatics analysis, Anp32b, Myl12a, and Nras were identified as potential R-loop-associated candidate gene signatures, and the functions of Myl12a were further verified in AP. These original findings fill a specific research gap in AP mechanistic study, and provide targeted, experiment-supported evidence for subsequent pathogenic mechanism exploration and therapeutic screening. Nevertheless, several limitations persist. First, the bioinformatics analysis results in this work were predominantly derived from public databases (GEO). Despite the correction of batch effects, sample heterogeneity among various study cohorts may still contribute possible bias. Second, this study did not include patients with AP of different etiologies (e.g., biliary, alcoholic, hyperlipidemic) or different severity grades for stratified analysis. Third, this study exclusively employed caerulein-stimulated AR42J cells as an *in vitro* model for AP, which can simulate the activation of proenzymes and inflammatory responses in acinar cells. While bioinformatic analyses pinpointed fibroblasts as key cells, acinar cell injury represents the initiating event of AP pathogenesis. Our biomarker Myl12a was also dysregulated in acinar cells; hence, we performed functional assays in this acinar cell system to probe its direct effects on acinar-intrinsic pathological phenotypes. Nevertheless, direct functional validation within fibroblasts was not conducted in the current work, which should be addressed in future studies. Fourth, among the three identified bioinformatic candidate gene signatures (Anp32b, Myl12a, and Nras), only Myl12a has undergone functional validation via knockdown *in vitro* and clinical sample exploration is also lack. The biological functions of Anp32b and Nras in AP, as well as their specific regulatory relationships with R-loop homeostasis, have not yet been confirmed through systematic overexpression or knockdown, as well as clinical experiments. Additionally, Nras were markedly up-regulated in bioinformatic datasets, while no significant changes were observed in the *in-vitro* AP model. Such inconsistent expression patterns of Nras may stem from biological differences between tissue-level cohort data and the simplified caerulein-induced AR42J cellular system. Tissue samples contain multiple heterogeneous cell subsets, where elevated Nras signals could originate from non-acinar cellular compartments. By contrast, the AR42J acinar cell model only reflects responses of a single cell type under *in-vitro* stimulation, which cannot recapitulate the complex multicellular milieu of intact pancreatic tissue, suggesting that its reliability as an AP target requires further evaluation in larger sample sizes and different AP models. Fifth, the mechanistic inferences in this study are based on association analyses between candidate gene signatures and R-loop scores as well as immune pathways; however, R-loop levels have not been directly measured, nor have the specific molecular mechanisms by which these gene signatures regulate R-loop homeostasis been elucidated. These limitations indicate that future studies should integrate multi-omics data, multiple AP animal models, and direct R-loop detection methods to further validate the functional mechanisms of these gene signatures and their clinical translational value.

In conclusion, this study systematically screened and identified key cell populations and candidate gene signatures linked to R-loop homeostasis imbalance in AP by integrating transcriptomic data with single-cell sequencing data, elucidating the potential mechanisms through which these molecules collaboratively regulate the onset and progression of AP via multidimensional immune-inflammatory pathways. These findings enhance our comprehension of the immune-inflammatory network and transcriptional dysregulation in AP, as well as establishing novel theoretical frameworks and molecular targets for early detection and targeted treatment of AP.

## Data Availability

The original contributions presented in the study are included in the article/**Supplementary Material**. Further inquiries can be directed to the corresponding author.
