# Supplementary material for: Integrative single-cell and bulk transcriptomic analysis identify R-loop-associated gene signatures driving immune-inflammatory networks in acute pancreatitis
Source: Front Immunol. 2026 Sep 29;17:1946699. doi: 10.3389/fimmu.2026.1946699 (PMC13657978; doi:10.3389/fimmu.2026.1946699)
Supplement: Supplementary file 1 [file DataSheet1.zip › Supplementary Tables/Supplementary Table 1.docx]

**Supplementary Table 1.** The details of AP-related datasets

| Dataset | Platform | Type | Species | Tissue | Sample number | Category |
| --- | --- | --- | --- | --- | --- | --- |
| GSE109227 | GPL6246 | RNA-seq | Musculus | pancreatic tissue | AP:control = 6:5 | Train cohort |
| GSE65146 | GPL6246 | RNA-seq | Musculus | pancreatic tissue | AP:control = 39:5 | Train cohort |
| GSE121038 | GPL10787 | RNA-seq | Musculus | pancreatic tissue | AP:control = 4:4 | Validation cohort |
| GSE3644 | GPL339 | RNA-seq | Musculus | pancreatic tissue | AP:control = 3:3 | Validation cohort |
| GSE181276 | GPL24247 | scRNA-seq | Musculus | pancreatic tissue | AP:control = 1:1 | / |
| GSE198183 | GPL24247 | scRNA-seq | Musculus | pancreatic tissue | AP:control = 1:1 | / |
