# Supplementary material for: Integrative single-cell and bulk transcriptomic analysis identify R-loop-associated gene signatures driving immune-inflammatory networks in acute pancreatitis
Source: Front Immunol. 2026 Sep 29;17:1946699. doi: 10.3389/fimmu.2026.1946699 (PMC13657978; doi:10.3389/fimmu.2026.1946699)
Supplement: Supplementary file 1 [file DataSheet1.zip › Supplementary Tables/Supplementary Table 3.docx]

**Supplementary Table 3.** RT-qPCR primers

| Gene | Primers (5’-3’) |
| --- | --- |
| Gapdh | F:5’-TGGTGAAGGTCGGTGTGAAC-3’ |
|  | R:5’-TTCTCAGCCTTGACTGTGCC-3’ |
| Anp32b | F:5’-TTCGGAGGTCTCGAGAGACTA-3’ |
|  | R:5’-AAATGCCTTCGCTTGCTTCG-3’ |
| Myl12a | F:5’-CCGAGCAGTATCCCCCTGAAG-3’ |
|  | R:5’-ATGCGCGTGAACTCGATGTA-3’ |
| Nras | F:5’-CACGAGCTGGCCAAGAGTTA-3’ |
|  | R:5’-TTCTTCATCCGGTACTGGCG-3’ |
| rat-Myl12a(ID:501203)siRNA-422 | F:5’-CCUGAGGACGUCAUCAGAAAUTT-3’ |
|  | R:5’-AUUUCUGAUGACGUCCUCAGGTT-3’ |
| rat-Myl12a(ID:501203)siRNA-186 | F:5’-CCAACGUGUUCGCCAUGUUUGTT-3’ |
|  | R:5’-CAAACAUGGCGAACACGUUGGTT-3’ |
| rat-Myl12a(ID:501203)siRNA-333 | F:5’-ACCUGGACGCCAUGAUGAAUGTT-3’ |
|  | R:5’-CAUUCAUCAUGGCGUCCAGGUTT-3’ |
